# OBSERVATION OF NEONATAL BEHAVIOR: CROSS-CULTURAL ADAPTATION OF THE NEWBORN BEHAVIORAL OBSERVATIONS

**DOI:** 10.1590/1984-0462/;2018;36;1;00017

**Published:** 2017-10-30

**Authors:** Marina Aguiar Pires Guimarães, Claudia Regina Lindgren Alves, Ana Amélia Cardoso, Lívia de Castro Magalhães

**Affiliations:** a Universidade Federal de Minas Gerais, Belo Horizonte, MG, Brasil.

**Keywords:** Translating, Mother-Child relations, Child development, Infant behavior, Tradução, Interação Mãe-Filho, Desenvolvimento infantil, Comportamento do lactente

## Abstract

**Objective:**

To conduct the cross-cultural adaptation for Brazilian Portuguese of the Newborn Behavioral Observations (NBO), a new resource for observing neonatal behavior and sharing information with parents.

**Methods:**

Methodological study of translation and cultural adaptation of the NBO system, which includes the Recording Form, with 18 items, the Recording Guidelines, with instructions to score each item, the Summary Form, to record suggestions based on the observation, and the Parent Questionnaire, to record the parents’ experiences. The adaptation process followed international recommendations for cross-cultural adaptation of health care protocols, which included requesting permission from the authors, translation, back translation and pre-test, followed by external evaluators who scored the quality of the adaptation, which was analyzed quantitatively. The quality of the adaptation of the instruments’ items was evaluated by the index of agreement between evaluators for conceptual and cultural equivalence.

**Results:**

Expert panel evaluation showed that the cross-cultural adaptation of the NBO protocols was both well understood conceptually and culturally appropriate, with 140 (77.8%) items presenting concordance index higher than 90% for conceptual and cultural equivalence. Items that did not reach adequate level of agreement were revised according to experts’ suggestions.

**Conclusions:**

The Brazilian version of the NBO system can be safely used, since the methodology was rigorous enough to ensure equivalence between the original and translated versions. The NBO should be tried in clinical practice, as it can contribute to improve the quality of maternal and child care.

## RESUMO

**Objetivo:**

Realizar a adaptação transcultural para a língua portuguesa brasileira dos protocolos do sistema *Newborn Behavioral Observations* (NBO), novo recurso para observação do comportamento do bebê e compartilhamento de informações com os pais.

**Métodos:**

Estudo metodológico de tradução e adaptação transcultural do sistema NBO, que inclui o Formulário de Registro, com 18 itens, o Guia de Registro, com instruções para pontuação de cada item, o Sumário para os Pais, para registro de sugestões e orientações decorrentes da observação, e o Questionário de Pais, para avaliação da experiência. Foram seguidas as recomendações internacionais para adaptação transcultural de protocolos da área de saúde, o que incluiu solicitação de autorização para tradução aos autores, tradução, retrotradução e pré-teste, seguido de avaliação externa por painel de especialistas, que avaliou a qualidade da adaptação de cada item, com cálculo do índice de concordância entre os avaliadores quanto à equivalência conceitual e cultural.

**Resultados:**

A avaliação do painel de especialistas evidenciou que a versão adaptada transculturalmente do sistema NBO foi bem compreendida conceitualmente e adequada culturalmente, com 140 (77,8%) itens apresentando índice de concordância maior que 90% quanto à equivalência conceitual e cultural. Itens que não obtiveram níveis adequados de concordância foram revisados conforme sugestões dos especialistas.

**Conclusões:**

A versão brasileira do sistema NBO pode ser utilizada com segurança, já que a metodologia empregada foi rigorosa, garantindo equivalência entre o original e a tradução. O sistema NBO está pronto para ser usado clinicamente, podendo contribuir para a melhora de qualidade da assistência materno-infantil.

## INTRODUCTION

The use of standardized instruments for the evaluation of infant behavior and development is urgent. Given the cost, the time and the expertise necessary to create new instruments, there is great interest in the use of resources created in other countries, which leads us to matters of language and cultural differences, and to the different concepts related with the cross-cultural use of evaluation instruments.^1^ ^,^ ^2^ The cross-cultural adaptation of an instrument aims at obtaining equivalence of content between the original and the translated version. This process involves both the translation and the adaptation of all items composing the original version, the instructions of application and response options, as well as the analysis of the measurement properties of the new version and the collection of normative data, when appropriate.^3^

There is no consensus regarding the best method of cross-cultural adaptation.^4^ ^,^ ^5^ Epstein et al.^5^ identified 31 different methods, but there is no evidence of superiority of a specific methodology. However, regardless of the process to be used, the methodology should be rigorous enough to guarantee as much accuracy in equivalence between the original and the translation^5^ and ensure reliable and valid measures.^6^

The Newborn Behavioral Observations (NBO)^7^ is a system used to observe behaviors, created to describe the competences and individualities of the newborn for the parents. The objective is to strengthen the parent-child relationship and to promote the development of a supporting relationship between the professional and the family. The NBO is a result of years of research and clinical practice with the Neonatal Behavioral Assessment Scale (NBAS)^8^, and consists of a simplified scale that maintains the conceptual richness of NBAS; however, it changes the focus of the diagnosis of neurobehavioral alterations towards the observation of potentialities, providing an experience of immediate connection between the infant and the parents.^7^ The NBO was created in English,^7^ but there is already a translation to Spanish.^9^

There are still few studies using the NBO, but evidence indicates that the system helps parents to understand the behavior of their children better, contributing to strengthen the connection and the interaction between mothers and newborns^10^ ^,^ ^11^, reducing maternal depression,^12^ besides facilitating the professional-family interaction^13^ ^,^ ^14^ and increasing the perception of trust among professionals regarding their work with newborns.^15^

The NBO fits in humanization strategies predicted in public health policies, such as the Kangaroo mother care,^16^ whose technical manual^17^ approaches the behavior and individuality of the infant, the signals of approximation and retraction, the neurobehavioral and psycho emotional development of the newborn and the mother-child connection. The NBO is an important tool for the recognition and decoding of these signals, giving the neonate a voice, facilitating both the effective mother-child communication and the establishment of a partnership between the family and the health professional.

Due to the relevance of using standardized resources that are easy to apply, to support the establishment of the initial connection between mother-child-health team and the absence of this type of resource in Brazil, the objective of this article was to perform the cross-cultural adaptation of the NBO system to Brazilian Portuguese.

## METHOD

Methodological cross-cultural adaptation study of the NBO, protocol for infant observation, with duration of approximately five to ten minutes. It can take longer, depending on the objective and the questions that come up during the administration. The NBO session is made at the presence of the parents, who are stimulated to participate in the observation. The protocol can be applied to infants aged up to three months of corrected age. It can be administered in a hospital, clinic, doctor’s office, early intervention programs and in the household. The necessary material to make the observation is simple and easily reproducible, consisting of a rattle, a red ball, a flashlight, the form and the registration guide.^7^ The theoretical reference of the instrument - detailed instructions for use, with information about the observational procedures, origin, and evolution of each item - is presented in the NBO manual, ^7^ of which only the standardized protocols composing the observational procedures were translated. It includes the Recording Form and the Recording Guidelines. The Summary Form and the Parent Questionnaire, which were also translated, were created afterwards and are not present in the manual.

The Recording Form is composed of 18 items of behavior and reflexes distributed in four dimensions, represented by the acronym AMOR: (A) autonomic responses (one item) - skin color, respiratory pattern and visceral function; (M) motor responses (seven items) - arm and leg, shoulder and neck tonus, level of motor activity, crawling response, reflections of suction, search and palmar prehension; (O) organization of status (five items) - ability to get used to stimuli and protect sleep, amount of crying, consolability and transition between the states of alert; (R) responsiveness (five items) - ability to stay alert and interact with people and objects, ability to interact with people and respond to visual and auditory stimuli. Each item is scored in a three-point scale, with specific criteria per domain, however, “3” is always the best response, and “1”, the worst. ^7^

Besides the items, the Recording Form of the observation includes the Anticipatory Orientation List, in which the areas that require more attention, orientation and follow-up can be pointed out, therefore serving as a guide between the professional and the parents. At the end of the form there is room for the professional to make a summary of the infant’s profile, according to the AMOR dimensions, and register recommendations, challenges, areas that need support and additional comments. ^7^ The score in the form is made according to the Recording Guidelines, which contain a brief description, specifying the response criteria for each item. ^7^

The Summary Form is a specific form to make a brief report of the infant profile, also organized according to the dimensions of AMOR. It includes recommendations, challenges, areas that need support and additional comments about the strong aspects of the newborn. This form is handed to the parents so that they can have a record of the observation. Even though the Summary went through the translation, back-translation and pre-test processes, it was not sent to the committee of experts because its elements are contained in the other NBO protocols.

The Parent Questionnaire was created to assess the perception of parents about the NBO session, and how the combined observation helped them to understand the infant’s behavior better.^10^ The Questionnaire has ten questions, divided in two parts:

1. six questions, and the three first ones are about the knowledge of parents regarding the newborn’s behavior. The others relate to the experience or perception of parents about the NBO session;
2. four questions about maternal data (date of birth, if this is the first child, schooling and language spoken in the household).

The questions in the first part are scored in a four-point Likert scale, except for the second and third questions, which are scored in a “1” (low) to “10” (high) scale.^18^

Initially, Dr. Kevin Nugent, author of the NBO, was contacted. He authorized the translation. The cross-cultural adaptation process was performed according to the stages recommended by Beaton et al.,^3^ with adaptations according to Epstein et al.^5^ Firstly, two health professionals fluent in English, but whose mother tongue is Portuguese, performed the independent translation of the original instruments. Both translations were analyzed by a committee composed of one of the translators and the research coordinator, who is also fluent in English. A synthesis was conducted to produce one single translation. The back-translation of the unified translation was executed by a professional translator, whose mother tongue was English, and then compared to the original version. Some adjustments were made to compose the pre-test version, which was sent to a multiprofessional team formed by 18 professionals who participated in an NBO certification course, ministered in Brazil by the Brazilian Institute, to be applied in at least five mother-child binomials. All of them were instructed to assess, informally, the quality of the translation. The professionals only requested the division of the item “arm and leg tonus” into “arm tonus” and “leg tonus”. Then, the revised version was sent to a committee of experts composed of ten health professionals (Table 1) fluent in English, with more than five years of experience in the field of infant development, to assess the quality of the translation.

**Table 1:** Characterization of the committee of experts.

Professional	Occupation	Title	Type of work	Experience (years)
P1	Occupational Therapist	PhD	Teaching and clinic	34
P2	Neuropediatrician	PhD student	Teaching and clinic	21
P3	Occupational Therapist	Master’s student	Clinic	5
P4	Physical therapist	PhD	Teaching and clinic	29
P5	Psychologist	PhD student	Clinic	12
P6	Occupational Therapist	PhD	Teaching and clinic	12
P7	Occupational Therapist	PhD	Teaching and clinic	12
P8	Physical therapist	PhD	Teaching and clinic	11
P9	Occupational Therapist	PhD student	Teaching	9
P10	Occupational Therapist	PhD student	Teaching	7

Tables containing the original items and their translation were sent to the committee, which analyzed the conceptual and cultural equivalence of each item. The conceptual equivalence refers to the meaning of the word, and the pertinence of the concepts in the original instrument and in the culture that is the target of the new version should be assessed. The cultural equivalence concerns the pertinence of the terms used in the translated version, and its understanding and comprehension should be analyzed in the cultural context of the population in which the instrument will be used.^3^ The experts of the committee used two criteria - “agree” and “do not agree” - to score the conceptual and cultural equivalence of each item of the questionnaires, besides the header, with data of identification and instructions, in a total of 180 items to be assessed. In cases of disagreement, the professional was invited to suggest changes in the translation, and was also able to suggest improvements, whenever pertinent.

The data were analyzed with the Statistical Package for the Social Sciences (SPSS), version 19. To assess the quality of the adaptation of the instrument, the percentage of agreement was calculated for each evaluator with the translation proposed regarding the conceptual and cultural equivalence. The percentage of agreement for each item and type of equivalence was calculated by multiplying the number of participants who agreed by one hundred, and dividing it by the total number of participants. The agreement rate between the evaluators considered as acceptable is 90%,^19^ and the suggestions made by the members in the committee were used to revise items with agreement below this level. All suggestions from the committee of experts were analyzed, and the adjustments were performed. Even in items considered to have good quality, when the suggestion contributed to facilitate the understanding in Portuguese, it was accepted. On the other hand, in some situations the suggestions of correction made by the committee were not accepted. After the discussion of the researchers, based on the original instructions manual of NBO, and consulting with the author of the instrument, the conclusion was that the corrections suggested were not consistent with the theoretical model of the NBO.

This study was approved by the Research Ethics Committee of Universidade Federal de Minas Gerais (COEP/UFMG) (CAAE: 29437514.1.0000.5149).

## RESULTS

The evaluation of the panel of experts showed that the cross-cultural adaptation of the instruments in the NBO system was well understood both conceptually and culturally. About 78% of the items presented concordance index (CI) higher than 90% in the conceptual and cultural equivalences. The Recording Form of NBO is formed by 81 items, of which 54 had 100% of concordance in the conceptual and cultural equivalence, 14 presented CI higher than 90% - of which 7 were adjusted - and 13 showed CI below 90%. OF these, eight were not modified, and five were modified according to the experts’ suggestion (Table 2).

**Table 2:** Example of items in the Recording Form of the *Newborn Behavioral Observations* revised due to the low percentage of concordance as to the equivalence in translation.

Original	Translation	Suggestion	Modification	Percentage of concordance as to the equivalences (%)	Justification
Tone	Tônus muscular	Tônus	-	Conceptual - 80.0 Cultural - 8.,9	Modification not performed for clarity
Weight	Peso de nascimento	Peso atual Peso ao nascimento	-	Conceptual - 70.0 Cultural - 90.0	The authors of the *Newborn Behavioral Observations* confirmed it is weight at birth
Parity	Paridade	Número de gravidez Número de filhos	-	Conceptual - 90.0 Cultural - 80.0	Authors of the *Newborn Behavioral Observations* reported it refers to parity (multiparous or primiparous)
Sleep protection	Proteção do sono	Sem sugestão	-	Conceptual - 90.0 Cultural - 80.0	Term maintained, because it is consistent with the contexto of the *Newborn Behavioral Observations*
Crawling response	Resposta ao engatinhar	Resposta de rastejar Reflexo de retirada Reflexo de engatinhar Resposta de engatinhar	Resposta de engatinhar	Conceptual - 60.0 Cultural - 77.8	Chanding “ao” to “de” accepted. The author of the *Newborn Behavioral Observations* confirmed that the ter mis “engatinhar”
Not stressed	Não	Não estressado	Não estressado	Conceptual - 100 Cultural - 80.0	Suggestion accepted
Needs support	Quantidade de suporte	Necessidade de suporte	Necessidade de suporte	Conceptual - 30.0 Cultural - 80.0	Suggestion accepted
Antecipatory guidance checklist	Lista de orientação antecipada	Lista de orientação antecipatória *Checklist* de orientação antecipada	Lista de orientação antecipatória	Conceptual - 75.0 Cultural - 62.5	Suggestion accepted

Note: only a few items are presented in this table; the sheet with all revised items can be obtained from the authors.

To prevent repeated items in the evaluation tables by the experts, the ones that were already in the Recording Form were not reproduced in the Recording Guidelines, resulting in 49 items, of which 24 had CI equal to 100%; 12, CI higher than 90%, of which 5 were adjusted; and 13, CI lower than 90%, of which 4 were not altered, and 9 were altered (Table 3). Examples of non-alteration are the items “The states are well defined, robust and easy to read, and/or state transitions are mild and predictable” and “Incapable of maintaining well-defined states; the transitions are unpredictable, abrupt and difficult to read”, which obtained 100 and 80% CI in conceptual and cultural equivalences, respectively, and in which the change in the term “read” to “observed”, “understood” or “perceived” was suggested. However, since the authors of the NBO^7^ refer to the observation as a reading of the newborn behavior (“*to read*”), we chose to maintain the consistency with the original.

**Table 3:** Examples of items in the Recording Guidelines of the *Newborn Behavioral Observations* revised due to the low percentage of concordance as to the equivalence in translation.

Original	Translation	Suggestion	Modification	Percentage of concordance as to the equivalences (%)	Justification
Minimal or no head turning	Não vira ou vira minimamente	Pouco vira ou não vira a cabeça Não vira a cabeça ou vira pouco a cabeça Não vira ou vira minimamente a cabeça	Não vira ou vira minimamente a cabeça	Conceptual - 44.4 Cultural - 70.0	Suggestion accepted
Initiates and maintains a modulated rhythmic suck	Inicia e mantém a sucção modulada e rítmica	Inicia e mantém ritmo modulado da sucção. Inicia e mantém uma sucção rítmica modulada Sucção rítmica modulada	Inicia e mantém sucção rítmica modulada	Conceptual - 70.0 Cultural - 90.0	Suggestion accepted
Clear-cut grasp-like movement	Movimentos evidentes de garra	Movimentos claramente evidentes de garra Movimentos evidentes de agarrar	-	Conceptual - 70.0 Cultural - 90.0	Consistent with the original
Unable to maintain well-defined states; transitions are unpredictable, abrupt, and difficult to read	Incapaz de manter estados bem definidos; as transições são imprevisíveis, abruptas e difíceis de ler	Incapaz de manter estados bem definidos; as transições são imprevisíveis, abruptas e difíceis de perceber	-	Conceptual - 100 Cultural - 80.0	Consistent with the original

Note: only a few items are presented in this table; the sheet with all revised items can be obtained from the authors.

The Parent Questionnaire of the NBO is constituted of 50 items, of which 28 presented with CI equal to 100%; 8 had CI higher than 90%, and 3 were adjusted; and 14 had CI below 90%, of which 10 were modified (Table 4). The item referring to the language spoken in the household was not altered, even though CI was 70% in the conceptual equivalence, and 90% in the cultural equivalence. Since in Brazil the language mostly spoken is Portuguese, the adaptation had to be performed, even though the correct translation of the original was “English”. The items “Before observation” and “After observation” obtained CI of 88.9 and 88.9% in the conceptual equivalence, and 77.8 and 88.9% in the cultural equivalence, respectively, and both were modified to “session”, after the evaluators’ suggestion.

**Table 4:** Examples of the items in the Parent Questionnaire of the *Newborn Behavioral Observations* revised due to the low percentage of concordance as to the equivalence in translation.

Original	Translation	Suggestion	Modification	Percentage of concordance as to the equivalences (%)	Justification
Some	Bastante	Mais ou menos / Algum Razoável / Um pouco	-	Conceptual - 55.6 Cultural - 75.0	Change suggested is equal to the other response criteria.
On the scale below, please give yourself a score for how much you knew about your baby’s behavior BEFORE the *Newborn Behavioral Observations* session, 1 meaning “very little” and 10 meaning “a lot”. (Place an X on the scale below)	Na escala abaixo, por favor, indique o quanto você sabia sobre o comportamento do seu bebê ANTES da sessão de *Newborn Behavioral Observations*, 1 significa “muito pouco” e 10 significa “muito”. (Marque um X na escala abaixo.)	Na escala abaixo, por favor, indique o quanto você sabia sobre o comportamento do seu bebê ANTES da sessão de *Newborn Behavioral Observations*, considerando que 1 significa “muito pouco” e 10 significa “muito”. (Marque um X na escala abaixo.)	Na escala abaixo, por favor, dê uma nota para o quanto você sabia sobre o comportamento do seu bebê ANTES da sessão de *Newborn Behavioral Observations*, considerando que 1 significa “muito pouco” e 10 significa “muito”. (Marque um X na escala abaixo.)	Conceptual - 100 Cultural - 88.9	Suggestion accepted
Now please give yourself a score for how much you know about your baby’s behavior AFTER the *Newborn Behavioral Observations* session, 1 meaning “very little” and 10 meaning “a lot”.	Agora, por favor, indique o quanto você sabe sobre o comportamento do seu bebê DEPOIS da sessão de *Newborn Behavioral Observations*, 1 significa “muito pouco” e 10 significa “muito”. (Marque um X na escala abaixo.)	Agora, por favor, dê uma nota para o quanto você sabe sobre o comportamento do seu bebê DEPOIS da sessão de *Newborn Behavioral Observations*, 1 significa “muito pouco” e 10 significa “muito”. (Marque um X na escala abaixo.)	Por favor, dê uma nota para o quanto você sabe agora sobre o comportamento do seu bebê DEPOIS da sessão de *Newborn Behavioral Observations*, considerando que 1 significa “muito pouco” e 10 significa “muito”. (Marque um X na escala abaixo.)	Conceptual - 88.9 Cultural - 88.9	Suggestion accepted
After the session	Depois da observação	Depois dessa sessão Depois da avaliação	Depois dessa sessão	Conceptual - 77.8 Cultural - 88.9	Suggestion accepted

Note: only a few items are presented in this table; the sheet with all revised items can be obtained from the authors.

In Tables 2, 3 and 4, referring to the Recording Form, the Recording Guidelines and the Parent Questionnaire, it is possible to verify the original item, the translation, the percentages of conceptual and cultural concordance, the modifications or non-modifications and the respective justifications. Due to the large number of items (180), which led to very long tables, we only presented the ones that, despite having CI higher than 90%, suffered changes, and also those with index lower than 80%, except for the ones already described.

## DISCUSSION

The methodology for the cross-cultural adaptation of the NBO instruments to Brazilian Portuguese used in this study demonstrated to be rigorous enough to ensure equivalence between the original and the translated version, with Cis above 90% in more than 2/3 of the items, generating instruments that can be used to guarantee the quality of the translation.

Because the cross-cultural adaptation is a developing area, there are still inconsistencies in the literature regarding the procedures to be conducted. There is great variability in the terminology used, and a lack of consensus about a systematized process for the evaluation of the equivalence between the original and the translated instrument.^20^ ^,^ ^21^ In this study, we chose to use the term “cross-cultural adaptation” instead of “translation”, because adaptation is a broader process, involving all aspects of the preparation of a test to be used in another language or culture.^1^ Even though there is no uniformity in the literature as to the best guideline for cross-cultural adaptation, ^5^ ^,^ ^20^ ^,^ ^21^ the process was rigorous, with a detailed description of each stage.

In terms of the process of adaptation, the choice was to follow the recommendations of Beaton et al.,^3^ recognized internationally, with some modifications. For example, only one back-translation was made, when the recommendation would be two. According to Epstein et al.,^5^ since there is no evidence of the superiority of a methodological strategy, it is not possible to recommend a single method. Therefore, the choice should be based on the preference and the logistics of the researchers, as well as on whatever seems to be most adequate in the context of the instrument. These authors suggest that the back translation is not mandatory, and that the committee of experts has an important role to guarantee the equivalence between the translated version and the original instrument. In this study, as recommended by Epstein et al.,^5^ we called highly qualified experts to assess the quality of the final version.

The committee of experts counted on professionals from many health areas, both researchers and physicians, all experienced in the field of infant development. Despite their expertise, only two of them were trained and certified by the Brazelton Institute to perform the NBO session, and this is a limitation of this study. Since this instrument is little publicized in Brazil, the theoretical base is not so known, and that generated some discrepancies in the process of equivalence evaluation (Table 4). For instance, the evaluators recommended the use of the word “evaluation” to refer to the NBO system, even though the NBO is an observational instrument, which does not aim at assessing and diagnosing problems in the behavior of newborns. If the committee was composed only by professionals trained in the NBO, items that require adjustments would possibly not be identified, as occurred in the pre-test, when we did not receive suggestions of correction in the translation. That happened because the professionals knew the items well, and probably did not pay that much attention to the writing. On the other hand, due to the practical character of the pre-test, we had to make an adaptation on the scoring criteria of the item arm and leg tonus, which was considered impossible to score without dividing it in arm tonus and leg tonus. The adaptation was accepted by the authors, who considered the possibility of incorporating it to the original protocol. Another example was the term “reading”, in the sense of reading the infants’ signs, which was maintained (Table 3), because it originally refers to the reading of a text. The language used in the field is very specific, and we tried to maintain the original connotation.

Even though we did not follow all the steps described by Beaton et al.^3^ strictly, the process was detailed, and included one justification for every decision, always trying to maintain the consistency with the theoretical reference of the NBO. Some stages were inverted. For example: the pre-test came before the evaluation by the committee of experts, which may be considered as a study limitation. However, this had a positive impact on the quality of the translation, because the panel worked with protocols that had been experimented in the clinical practice. It is worth to mention that the NBO system is composed of observational items and questionnaires, which justifies adjustments in the cross-cultural adaptation methodology, as discussed by Epstein et al.^5^

There is a shortage of instruments that help parents and professionals to understand better the signals of the newborns, and the NBO is a specific tool for the recognition of these signals, facilitating the effective communication between parents and children. The NBO has the advantage of being brief, simple and easy to apply, which allows its use in different contexts, such as outpatient clinics, home care, hospitals and even neonatal intensive care units, with newborns at risk.^7^ The system can be a good complementation for the Kangaroo methods, since it helps mothers to read the signals of the infants and give professionals a tool to initiate positive relationships with the parents. The Brazilian version of the NBO should be applied at the clinic and in studies to verify its validity and adaptation to the Brazilian context.
